# Gravid Spot Predicts Developmental Progress and Reproductive Output in a Livebearing Fish, *Gambusia holbrooki*

**DOI:** 10.1371/journal.pone.0147711

**Published:** 2016-01-25

**Authors:** Nor Hakim Norazmi-Lokman, G. J. Purser, Jawahar G. Patil

**Affiliations:** 1 Fisheries and Aquaculture Centre, Institute for Marine and Antarctic Studies, University of Tasmania, Launceston, Tasmania, Australia; 2 Fisheries and Aquaculture Centre, Institute for Marine and Antarctic Studies, University of Tasmania, Hobart, Tasmania, Australia; 3 Inland Fisheries Service, New Norfolk, Tasmania, Australia; 4 School of Fisheries and Aquaculture Sciences, Universiti Malaysia Terengganu, Kuala Terengganu, Terengganu, Malaysia; University of Hyderabad, INDIA

## Abstract

In most livebearing fish, the gravid spot is an excellent marker to identify brooding females, however its use to predict progress of embryonic development, brood size, timing of parturition and overall reproductive potential of populations remain unexplored. Therefore, to understand these relationships, this study quantified visual attributes (intensity and size) of the gravid spot in relation to key internal development in *Gambusia holbrooki*. Observations show that the colour of the gravid spot arises from progressive melanisation on the surface of the ovarian sac at its hind margin, rather than melanisation of the developing embryos or the skin of the brooding mother. More importantly, the gravid spot intensity and size were closely linked with both developmental stages and clutch size, suggesting their reliable use as external surrogates of key internal developmental in the species. Using predictive consistency of the gravid spot, we also determined the effect of rearing temperature (23°C and 25°C) on gestation period and parturition behaviour. The results show that gestation period was significantly reduced (F = 364.58; df = 1,48; P˃0.05) at 25°C. However there was no significant difference in average number of fry parturated in the two temperature groups (P<0.05), reaffirming that gravid spot intensity is a reliable predictor of reproductive output. The parturition in the species occurred predominantly in the morning and in contrast to earlier reports, tails of the fry emerged first with a few exceptions of head-first, twin and premature births. This study demonstrates utility of the gravid spot for downstream reproductive investigations in a live-bearing fish both in the field and laboratory. The reproducibility of the relationships (intensity with both developmental stage and clutch size), imply that they are also relevant to wild populations that experience varying temperature climes and stressors, significant deviations of which may serve as indicators of environmental health and climate variability.

## Introduction

The eastern mosquitofish *Gambusia holbrooki* is one of the most widely introduced and invasive freshwater fish species in the world [1]. Closely related to the western mosquitofish *G*. *affinis* and often discussed together as Gambusia, the life history of both species is of interest due to their utility as models of viviparity, mosquito control, environmental pollution and of late as a threat to native aquatic biodiversity [2, 3]. Originally considered as worthless, Gambusia were regarded as effective and beneficial to control mosquito and hence translocated worldwide to control the spread of malaria [4–6] during the early to mid-20^th^ century. However they are now known as a pest and harmful to the native species in places of their introduction [7–14], needing urgent solutions for control [15]. Predominantly, most available biological information is restricted to *G*. *affinis* with a general assumption that *G*. *holbrooki* also has a similar biology and behaviour, often leading to confusion and incorrect inferences. For example, there are significant differences in terms of morphology, chromosome structure and genetic makeup [1], yet shared gamity (WZ/ZZ) between *G*. *holbrooki* and *G*. *affinis* based on information of the latter has been assumed even in recent times [16]. Systematic investigations therefore are necessary to resolve species-specific differences that are likely to yield novel insights into basic vertebrate biology, such as reproductive strategies and evolution as well as application of this knowledge to environmental pollution and management of invasive populations among others.

The detailed embryonic life stages of *G*. *affinis* have been described [17, 18] but not for *G*. *holbrooki*. As in other viviparous teleosts, fertilization in *G*. *holbrooki* occurs internally where fully matured oocytes are fertilized within the ovary by spermatozoa held following copulation in an intra-ovarian structure known as a ‘delle’ [18–20]. The embryos develop in the ovarian follicles during the gestation period and are born as juveniles [20] with fertilization of the next clutch thought to occur after the birth of the previous clutch [18]. Critically, the staging of embryonic development in the species necessitates sacrifice of brooding mothers precluding multiple time point observations (i.e. pre-and post-parturition). Most livebearers, including *G*. *holbrooki* also exhibit asynchronous embryonic development and parturition with overlapping and variable clutch size—often limiting an ability to predict reproductive output in wild fish or conduct experiments such as sex reversal in the laboratory.

Some Gambusia species also possesses an anal spot (a dark spot along the ventral midline close to the anal pore) that has been used as a tool to predict their reproductive cycle. Several studies on the life history of Gambusia have suggested that the size and pigment intensity of an anal spot can be linked to the female’s reproductive cycle—the spot becoming larger [21] and intensely pigmented [1, 22, 23] with advancing maturity. However, no study has reported the occurrence of an anal spot in *G*. *holbrooki* and our observation on the introduced Tasmanian population suggests that they do not possess the anal spot, suggesting the need for an alternative external surrogate to predict the reproductive status of a pregnant female.

Like most poecilid fishes, one of the distinct features that differentiate *G*. *holbrooki* females and males is the dark pigmented spot known as the gravid-spot (lateral and cranial to the anal/genital pore) possessed by mature females. While the gravid spot is an excellent marker to identify mature and brooding females its function and relationship to the *G*. *holbrooki* reproductive cycle has not been fully explored. As a first step towards understanding the origin and function of the gravid spot and to address limitations associated with developmental and reproductive investigations in this viviparous fish, we tested if the size and pigment intensity of the gravid spot could serve as an external and non-invasive indicator for developmental staging as well as to predict the timing of parturition and clutch size. Two different experiments were conducted in this study to achieve the objectives of: i) relationship between gravid spot and developmental progress; and ii) observation on gestation and parturition in this species. They were conducted sequentially where the results obtained in the first experiment were used to structure the second experiment, with the two rearing temperature regimes testing robustness/plasticity of the relationships.

## Materials and Methods

### Source of specimens

Wild fish collection, handling and transportation were carried out as stipulated under the Inland Fisheries Service Tasmania permit and where necessary fish were euthanased using Benzocaine. All procedures were also reviewed and approved by the University of Tasmania Animal Ethics Committee (AEC A12787). Stocks of *Gambusia holbrooki* used in this experiment were collected using dip nets in September 2013 from the Tamar Island Wetland Reserve (TWIR), Launceston, Tasmania. They were maintained in a recirculating aquaculture system (temperature: ±25°C; salinity: 0ppt; 16L:8D) at the Institute for Marine and Antarctic Studies (IMAS) Aquaculture Centre, University of Tasmania, Newnham, Tasmania. The fish were fed twice daily to satiation with commercial fish pellets (TetraMin^®^ tropical granules, Germany).

### Developmental staging

One hundred females displaying a range of sizes (20.0–50.0 mm SL) and maturity levels were chosen randomly, using the anal fin morphology as a guide to distinguish them from males. Prior to digital photography, fish were euthanased by an overdose of Benzocaine (1:2000). The fish were then dissected and the eggs/embryos counted, measured and staged. In the absence of detailed developmental staging in *G*. *holbrooki*, a set of morphological staging criteria as described for *G*. *affinis* [17], was adopted for this study and broadly categorised into five stages (I-V) based on the morphological criteria (Table 1). In cases where there was more than one distinct embryonic stage in a female, the stage with the highest number of embryos was assigned to the female for the purpose of analysing the relationship between gravid spot and developmental stages. Fecundity was measured as the total number of number eggs/embryos in each female (unfertilized eggs + embryos in each female) while clutch size was measured as the total number of embryos only.

**Table 1:** Criteria used for staging *G*. *holbrooki* embryos.

Developmental criteria*	Stage
Preliminary development of optic cupules/eyes.	I
Anterior neural tube broadened.	II
Eye pigmentation appears. Vitteline veins are visible.	III
Fine capillaries are developed on the surface of the pericardial sac. Appearance of melanophores on the tail. Gill slits are visible.	IV
Late developmental stage. First rays of the caudal fin become apparent. The mouth and nostrils are visible through the pericardial sac.	V

*Staging adopted from those described for *G*. *affinis* [17].

### Digital imaging of the gravid spot

Photographs of the fish were taken under natural white colour bulbs on a white surface, using a DSLR camera (Nikon D3000 equipped with 18-55mm f/3.5–5.6G VR DX AF-S Nikkor lens, Japan). The camera was set at a fixed distance of 25 cm above the sample using a tripod. A colour reference card and a ruler were included in each photograph to allow comparisons between different photographs/individuals. All images were saved in RAW format for visual analysis [24, 25]. Length of the fish, its gravid spot size (area) and spot intensity were measured using ImageJ software [26] in each image. The intensity values are represented within a range of 0 (minimum value, black) to 255 (maximum value, white) [27].

### Gestation and parturition

The gestation period was observed in fish exposed to one of two temperatures: 23±1 and 25±1°C. Temperature was maintained by using room air temperature controllers and was recorded daily. Fifty pregnant females were chosen based on the gravid spot intensity value (intensity range: 28–38; values established from the first experiment) and randomly divided into two groups and exposed to the two temperature regimes. Each fish was held in individual static tanks (2.5L; 0ppt; 16L:8D) fitted with a breeding trap to prevent cannibalism by the mother. The brooding fish were fed to satiation twice daily with commercial fish pellets (TetraMin^®^ tropical granules, Germany) and water changes were undertaken every two days to maintain water quality. Gestation period was measured as days between parturitions as exact timing of fertilization could not be ascertained.

Based on preliminary observations, parturition activity was closely monitored twice daily over a six month period: morning (0900-1100h) and evening (1500-1700h) and number of newborn fry were recorded at each time. The females were considered to cease reproducing if they did not show any signs of gestation such as swollen belly and when parturition did not occur for more than 50 days since the last parturition event. After parturition, the newborn fry were transferred to a separate rearing tank.

### Statistical analysis

The data were analysed statistically by using IBM SPSS Statistic software (version 22). One-Way ANOVA was used to determine differences between the gravid spot intensity and the assigned developmental stages. The data was tested for normality and Pearson’s correlation analysis was applied to determine the relationship between the following: the size of the gravid spot with the intensity, fecundity and total fish length; the intensity of the gravid spot with the fecundity and total fish length; and the fecundity and total fish length [28]. A multiple regression analysis to predict the clutch size with gravid spot size, its intensity and fish length as independent variables was also carried out.

## Results

### Gravid spot and embryonic development

Of the one hundred females examined, the presence of a gravid spot was observed in nearly all (92%) with intensity values ranging between 28–92. Developing embryos were found in 51 of these females while 39 possessed only mature eggs and 2 without any eggs. The total length of females without a gravid spot and eggs (n = 8) ranged between 20.0–27.0 mm while the total length of females with a gravid spot ranged between 30.9–50.0 mm.

**Fig 1:**
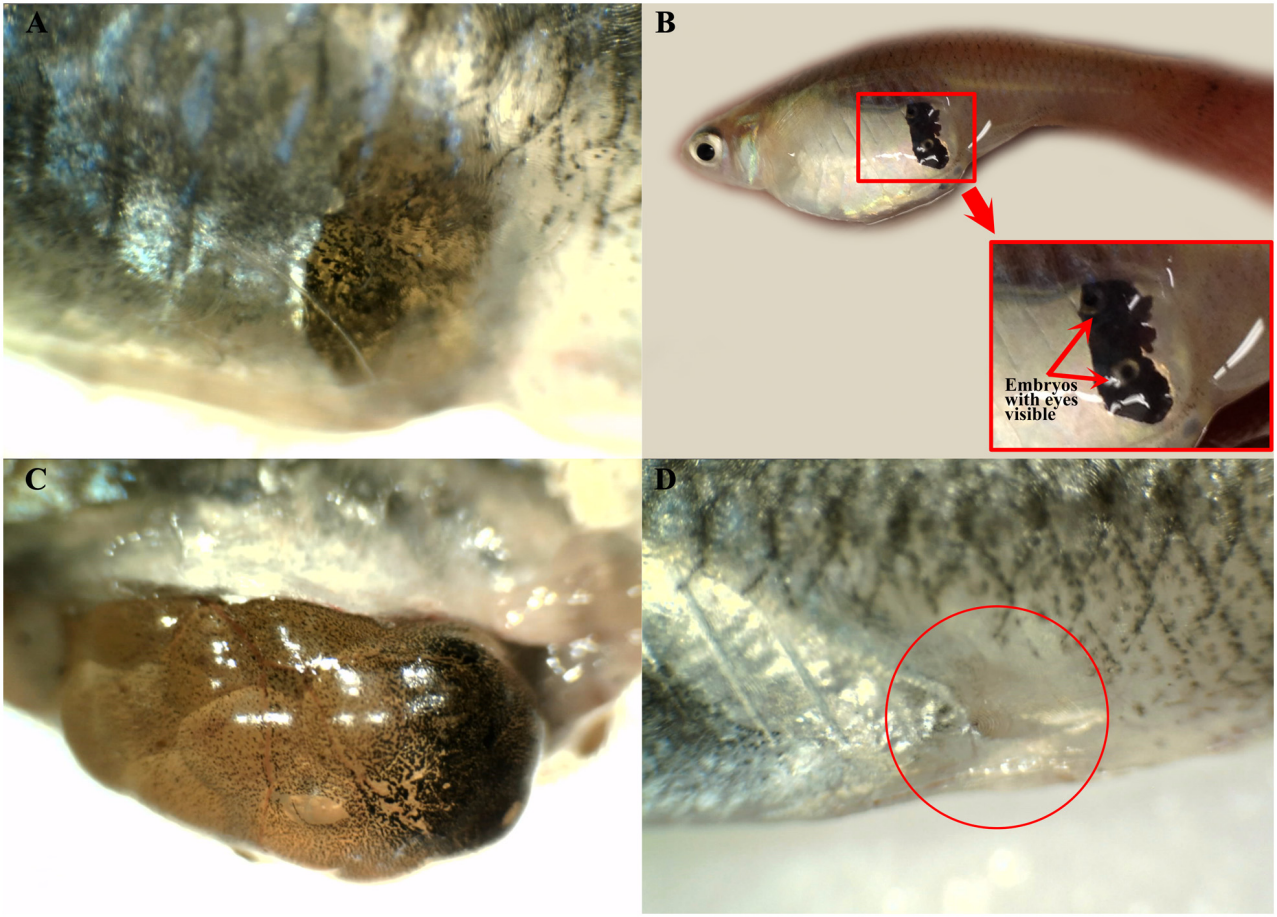
Gravid spot of *G*. *holbrooki*. (a) Gravid spot of a female *G*. *holbrooki* with high intensity value (less dark). The yellowish colour of the yolk/eggs can be seen when observed under dissecting microscope. (B) Embryo’s eyes at final developmental stage are visible through the gravid spot (red arrows). (C) Black pigment is scattered over the sac but is concentrated on the posterior margin of the ovarian sac. (D) Area of the skin corresponding to the gravid spot (circled red) appears as a translucent window after the ovarian sac was removed from the body cavity. Dorsal to the top and anterior to the left.

The gravid spot consisted of black pigments or melanophores that covered the ovarian sac. In females that possessed a gravid spot with a high intensity value (lighter/less dark), the eggs could be seen under a dissection microscope through the gravid spot (Fig 1a). In females with embryos at the final developmental stage, the eyes of the embryos were visible to the naked eye despite the black pigmentation (Fig 1b). Although the black pigment was scattered over the sac, it was most dense at the posterior margin of the embryonic sac, corresponding to the externally visible gravid spot (Fig 1c). After the ovarian sac was removed from the body cavity, the area of the fish skin corresponding to the gravid spot appeared translucent (Fig 1d).

### Embryonic Stages

**Fig 2:**
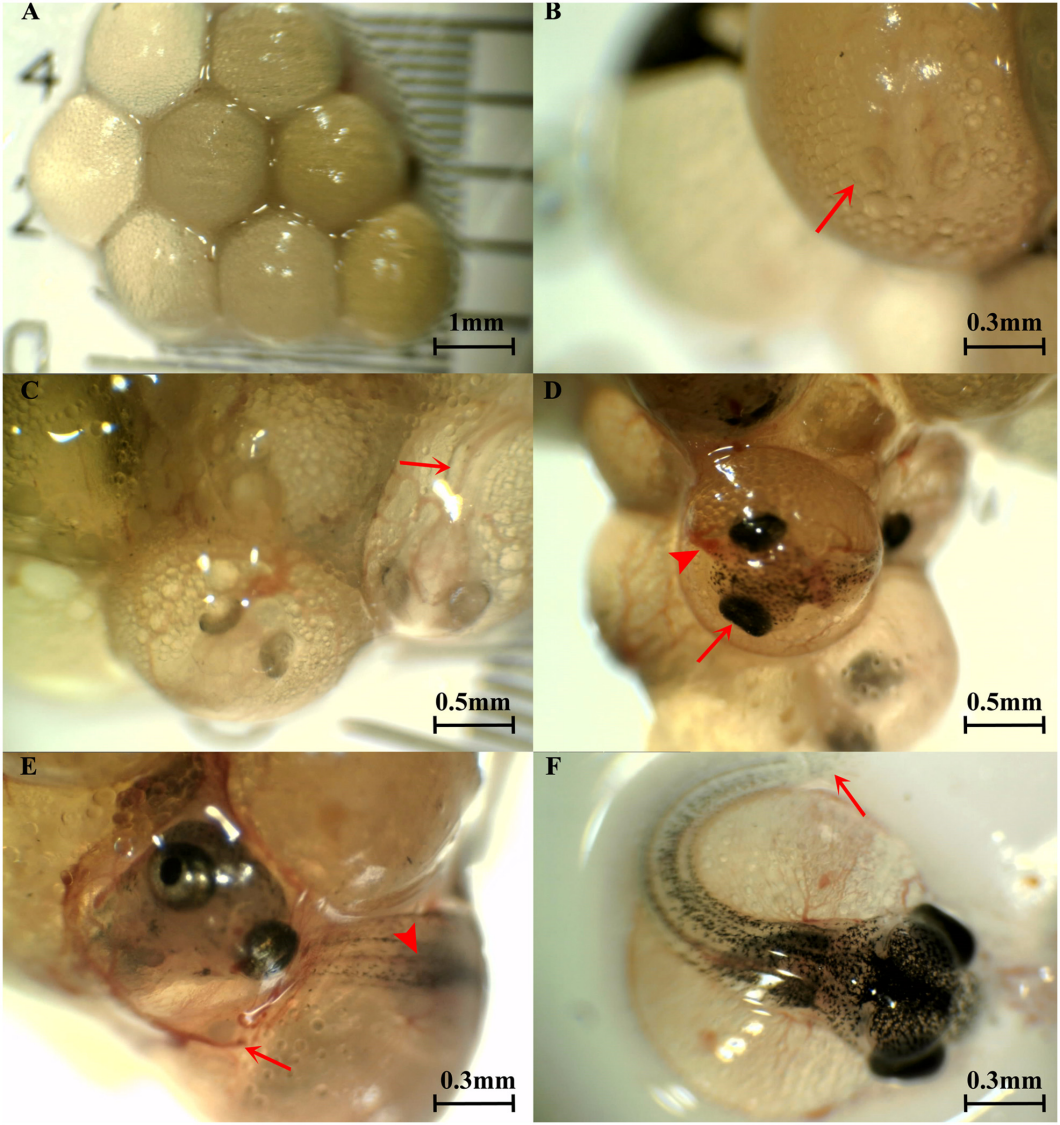
Panel showing mature eggs and embryos of *G*. *holbrooki* at various developmental stages. (A) Mature unfertilized eggs with no sign of cell division; (B) Stage I embryos with rudimentary optic cupules/eyes (arrow); (C) Stage II embryo with anterior neural tube broadened (arrow); (D) Stage III embryo with eye pigmentation prominent (arrow) and visible vitteline veins (arrow head); (E) Stage IV embryo with fine capillaries on the surface of the pericardial sac (arrow), melanophores on the tail (arrow head) and visible gill slits and (F) Embryo at late developmental stage (Stage V), showing the caudal fin rays (arrow). At this stage the mouth and nostrils are also visible through the pericardial sac [17].

Of the 92 females that possessed a gravid spot with eggs/embryo, 51 contained embryos or fertilized eggs. The number of embryos in each female examined ranged between 6 and 56 at different stages of development and fell into one of the five categories summarised in Table 2. Representative images including the unfertilised eggs are shown in Fig 2.

**Table 2:** Number of *G*. *holbrooki* females with overlapping developmental stages.

Stages	Stage I	Stage II	Stage III	Stage IV	Stage V
Stage I					
Stage II	6				
Stage III	14	1			
Stage IV	9	1	-		
Stage V	8	2	2	-	

Note, no fish carried embryos with more than two developmental stages, but all the fish had unfertilized egg”

**Fig 3:**
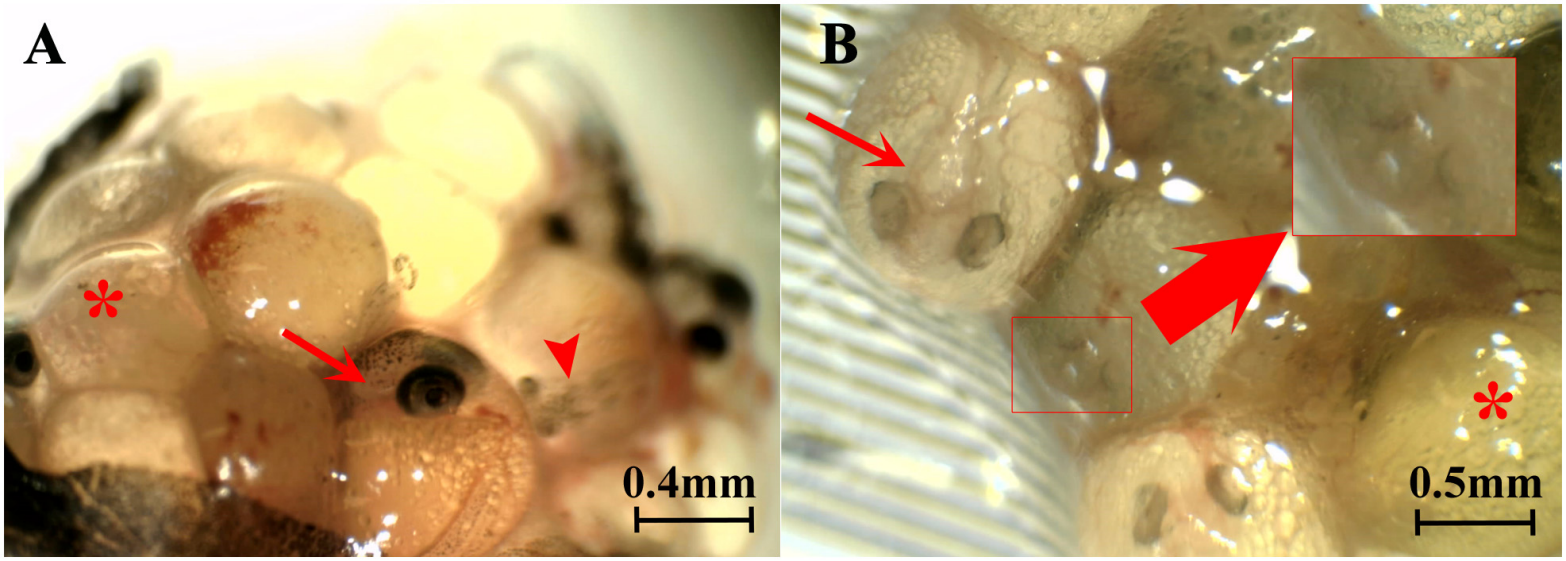
Dissected ovarian sac of two *G*. *holbrooki* females showing embryos at multiple stages of development suggesting superfetation. (A). A developed (stage IV; arrow) as well as an early stage (stage II; arrow head) embryo alongside mature unfertilized eggs (asterisk). (B) Stage II (arrow) and stage I embryos (inset) together with mature unfertilized eggs (asterisk).

The clutches of embryos from 43 (84.3%) females were at more than one developmental stage (Fig 3 and Table 2). Invariably, all females with embryos also contained mature unfertilised eggs. The number of females with their assigned developmental stage is presented in Table 2.

### Relationship of gravid spot intensity and size with embryonic stage and fish length

**Fig 4:**
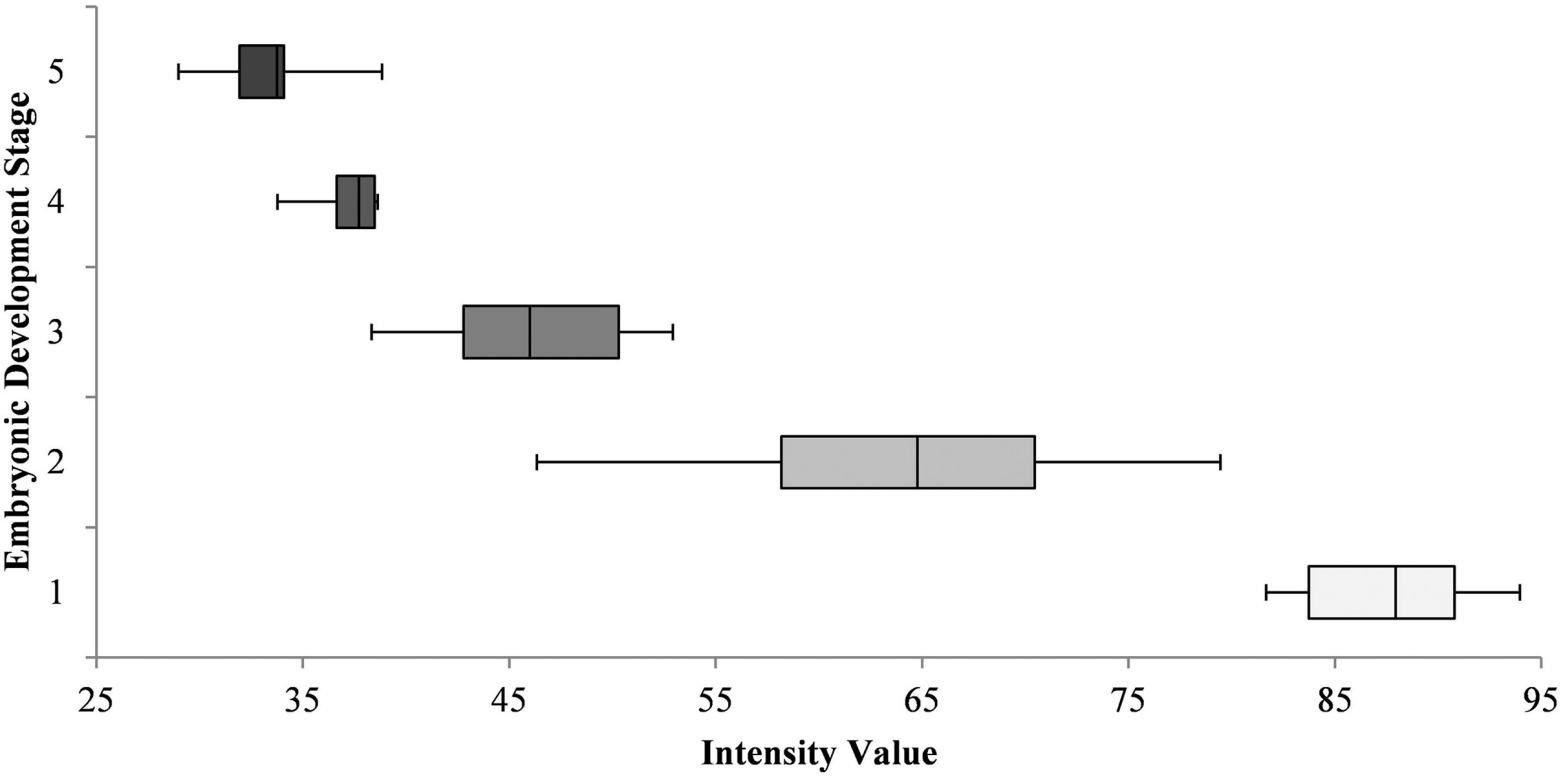
Range of gravid spot intensity values corresponding to fish with assigned embryonic stages of development. As fish carried more than one developmental stage (generally 2) they assigned to the most dominant developmental stage they carried.

The gravid spot intensity values observed in this study ranged between 28 (minimum) and 92 (maximum). Fig 4 illustrates the range of gravid spot intensity values associated with the observed embryonic developmental stages. One-way ANOVA analysis confirms that there is a significant difference (F = 127.42; df: 4, 47; P<0.05) between the intensity values corresponding to each embryonic stages of development.

**Fig 5:**
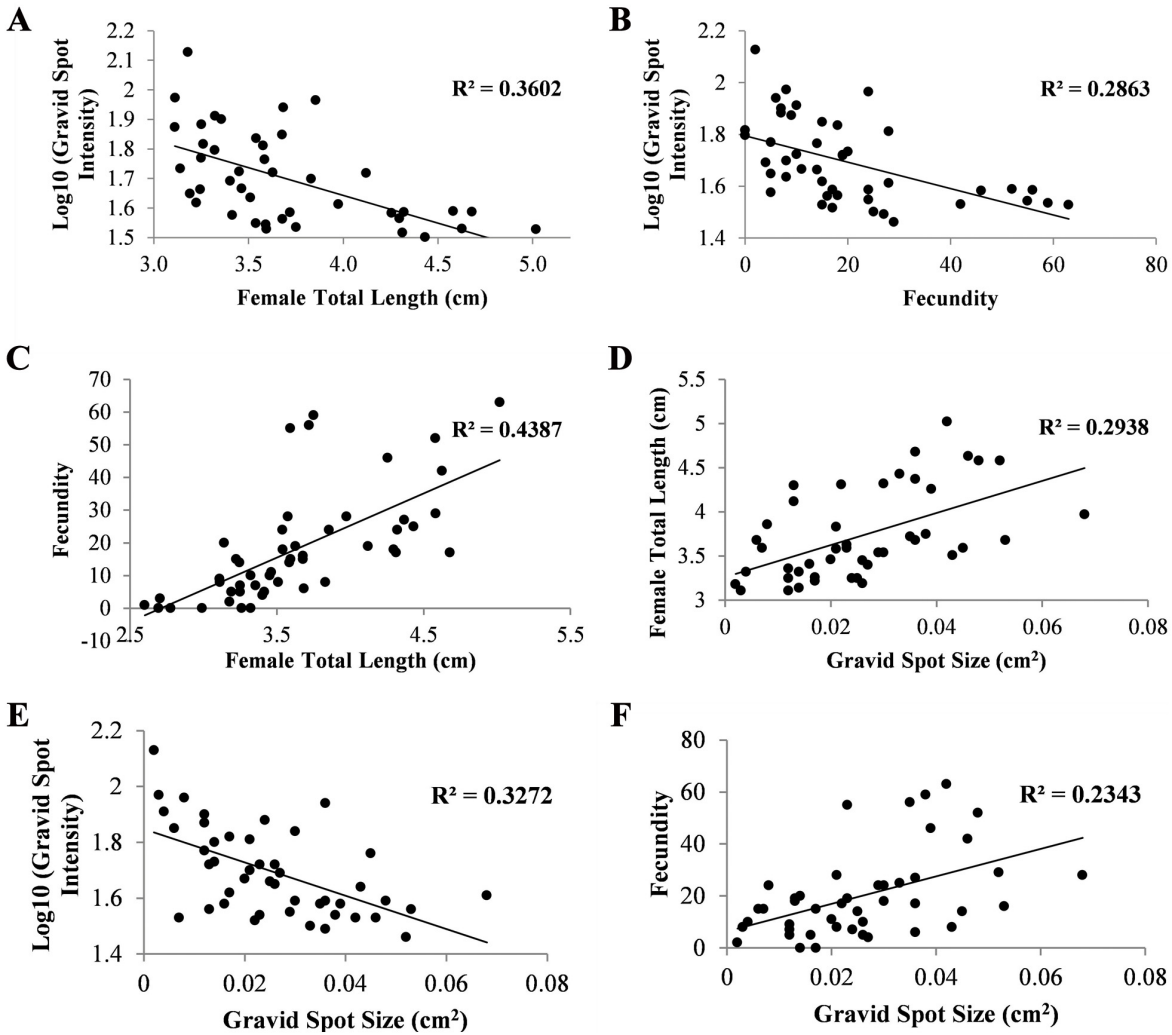
Relationship between (A) gravid spot intensity and female size (total length); (B) gravid spot intensity and fecundity; (C) fecundity and female size (total length); (D) female size (total length) and gravid spot size; (E) gravid spot intensity and gravid spot size and (F) fecundity and gravid spot size. Note: Higher and lower intensity values represent lighter and darker coloration respectively.

Pearson’s correlation analysis showed a significant relationship between the intensity of the gravid spot with the size of the females (r = -0.60, n = 92, P<0.01; Fig 5a) and fecundity (r = -0.54, n = 92, P<0.01; Fig 5b). The relationship between the fecundity and the size of the fish was also strong (r = 0.66, n = 100, P<0.01; Fig 5c). The sizes of the gravid spot increased with the length of the fish (r = 0.572, n = 92, P<0.01; Fig 5d), its intensity (r = -0.66, n = 92, P<0.01; Fig 5e) and fecundity (r = 0.48, n = 92, P<0.01; Fig 5f). In the multiple regression analysis, the assumptions of linearity, independence of error, homoscedasticity, unusual point and normality of residuals were met. These variables (spot size, its intensity, and fish length) collectively and reliably (F = 12.659; df: 3, 47; P<0.05) predicted the clutch size (CS):

CS=1.835 − (0.85 X SS)  +  (0.196 X SI)  + (3.543 X FL), where SS and SI are gravid spot size and intensity respectively, and FL is fish length. Regression coefficients and standard errors can be found in S1 Table.

### Gestation period and parturition

**Fig 6:**
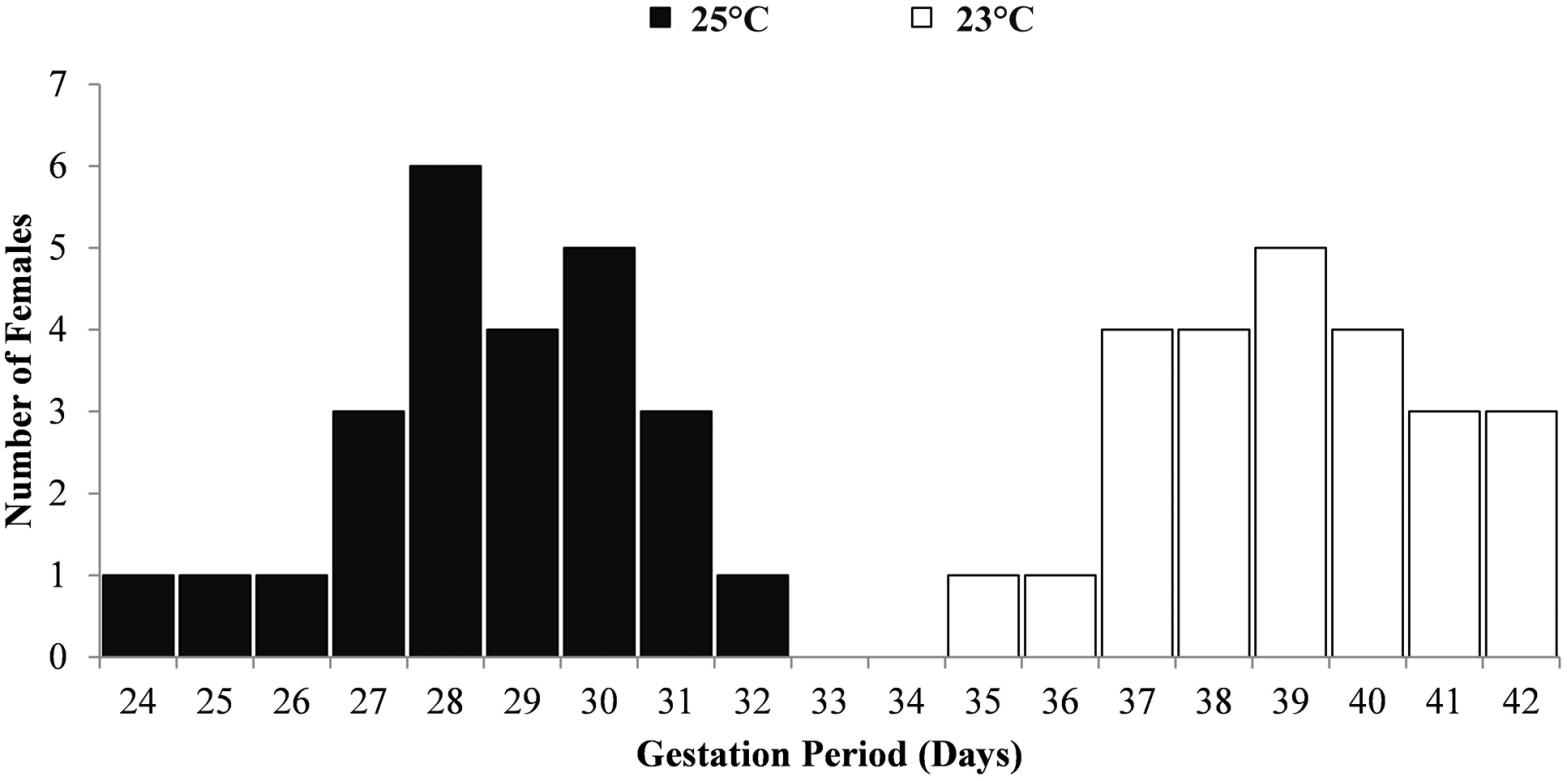
Gestation period of *G*. *holbrooki* at two different rearing temperatures.

The gestation periods of *G*. *holbrooki* females reared at two different temperatures are shown in Fig 6. As expected, the average gestation period (number of days) for females reared at 23°C was significantly (F = 364.58; df = 1,48; P˃0.05) longer (39±1.91 days) than those reared at 25°C (28.6±1.94 days), with clear range separation (Fig 6) with an almost identical and uncanny range of 9 and 8 days between the quickest and the slowest gestation event at the 25°C and 23°C temperatures respectively.

**Fig 7:**
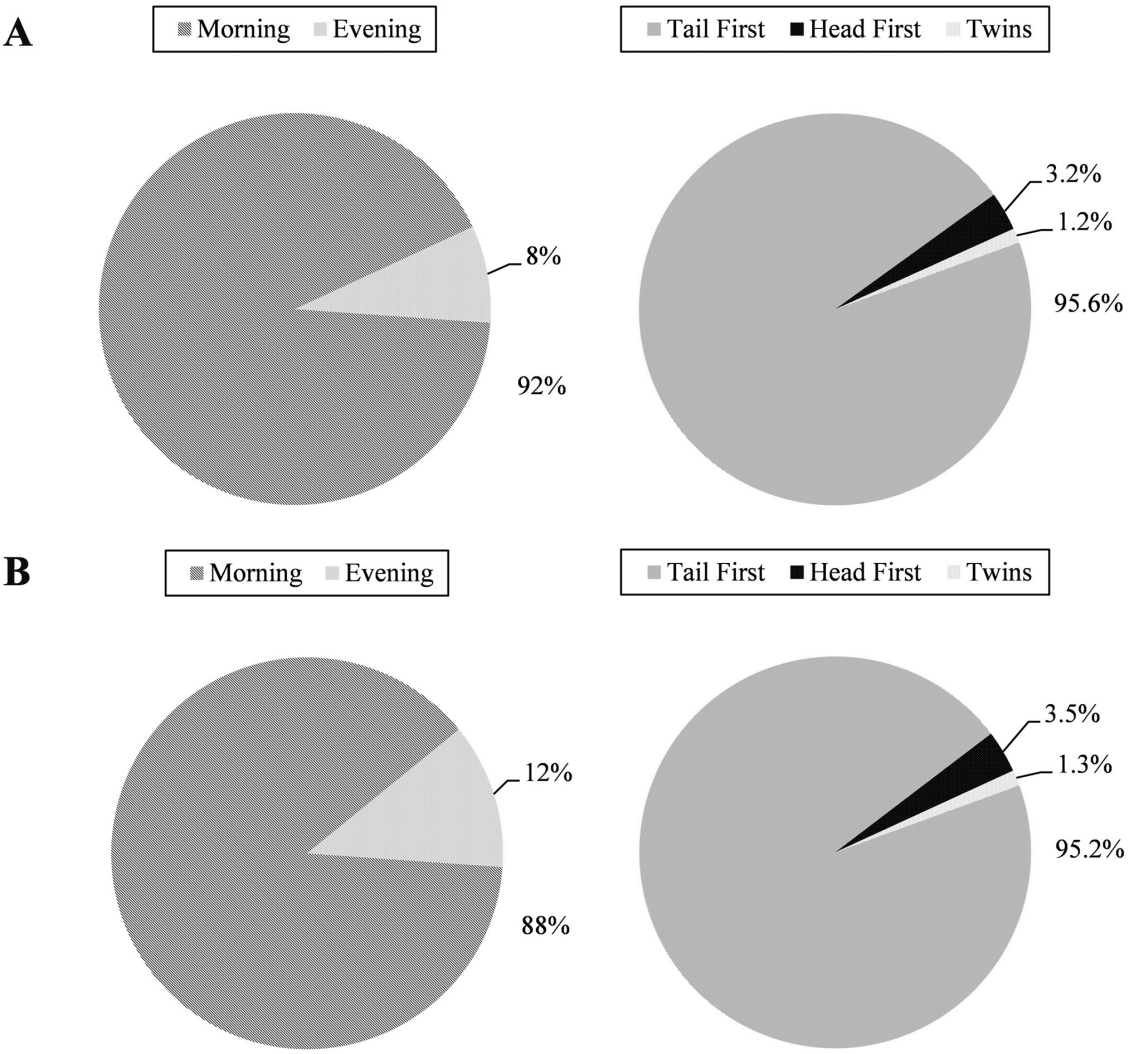
Frequency of parturition timing (left) and postures (right) in *G*. *holbrooki* at: (A) 25°C and (B) 23°C.

Based on observations, parturition in *G*. *holbrooki* occurred predominantly in the morning (0900-1100h) at both temperatures (Fig 7a and 7b). At 23°C, 22 out of 25 (88%) females parturated in the morning while the number was 23 (92%) for females reared at 25°C. The remaining females (3 and 2 at 23°C and 25°C respectively) parturated in the afternoon (1500-1700h). There were no significant differences (P<0.05) in timing (morning v/s evening) of birth between the rearing temperatures. Typically the duration of the parturition event ranged from 5 minutes up to 3hrs depending on clutch size, by which time all the fry in the clutch were delivered.

Three types of parturition postures were encountered. They were tail first; head first and twin births (see S1 Video). The frequency of occurrence for each parturition posture is shown in Fig 7a and 7b. Each of these events occurred randomly, for example, in one female, the first two fry were born tail first, whereas the third fry was born head first followed by tail first again for the 4^th^–11^th^ fry, followed by twins (2 fry released simultaneously). There were no differences (P<0.05) in the frequency of birthing postures between the two rearing temperatures.

**Fig 8:**
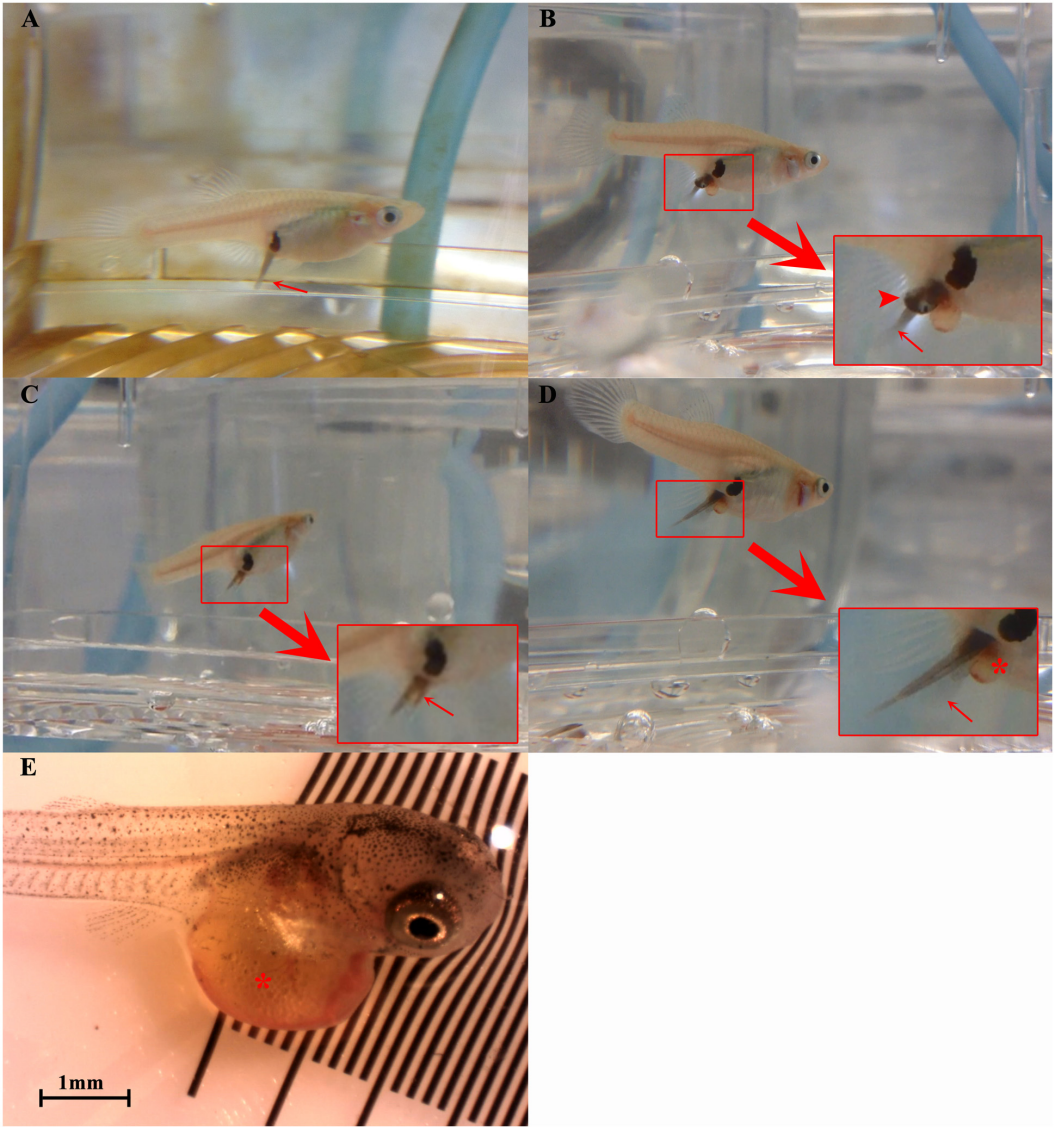
Panel summarizing the parturition process in *G*. *holbrooki*. (A) Most frequently observed “tail-first” parturition event where tail of the fry (red arrow) emerged first from the genital pore; (B) Infrequent ‘head-first’ parturition event where the head (arrow head) of a fry emerged first from the genital pore. In this example, note the tail (red arrow) of the next fry born emerged before the one ahead was completely released; (C) On few occasions ‘twin births’ where two fries could be seen emerging simultaneously out of the genital pore (red arrow); (D) Parturition event showing a fry being born with yolk (asterisk) still intact. Note, the free tail (arrow) with head still stuck in the ovarian sac; (E) A safely born fry with the yolk sac (asterisk) still intact. Also see S1 Video.

Representative photographs of the birthing posture are also presented in Fig 8. An event where the tail of newborn fry emerged first followed by one with a head-first is shown in Fig 8a and 8b respectively. Typically, the curled fry, in the sac, positioned itself at the opening of the genital pore followed by an uncurling action of the tail, expelling itself (tail) out of the genital pore. The head and hence the newborn was later released between 15 to 40 seconds after the emergence of tail, usually preceded by a wiggle of the tail. A few seconds later, the tail of the second fry emerged and the process continued till the last fry in the clutch was born. In some cases, the tail of the next fry to be born emerged before the one ahead was completely released from the mother (Fig 8b), with birth of two fry synchronised resulting in ‘twin births’ (Fig 8c) on odd occasions.

Commonly, the yolk sac was completely absorbed at the time of birth. However, in fry from three females (coincidentally all reared at 25°C), the yolk sac was still intact at the time of birth (Fig 8d). In these instances, the females showed signs of difficulty and stress during the parturition process as this lasted longer (5–10 minutes) than usual (15 to 40 seconds) and the survival of the babies was very poor, with only those delivered with yolk sac intact surviving (8e) while those where the yolk sac burst during the parturition event died instantly after birth.

## Discussion

The gravid spot has been known as an indicator of maturity in female livebearing fish such as Gambusia [21, 29] but its origin, function and relationship to the respective developmental and reproductive traits was previously not well-defined, particularly in *Gambusia holbrooki*. Discussed here are our observations on the source of gravid-spot pigmentation, its relationship to developmental progress and potential reproductive outputs in the species.

### Source of gravid spot colour

This study shows that the gravid spot derives its colour from the black pigments covering the ovarian sac. This was particularly obvious when the area (of the fish skin) corresponding to the gravid spot became colourless/translucent as soon as the ovarian sac was removed from the brooding fish. These observations in part support the ideas of Peden [30] who suggested that the gravid spot is ‘formed’ by the tearing of peritoneum in the abdominal cavity underneath the fish skin, exposing the egg/embryo sac and its pigments and expanding in size with maturity.

Our observations show that gravid spot colour is physically derived from the hind margins of the embryo sac, where its dark pigmentation is most concentrated and intensifies as the embryos advance in development. In contrast, the gravid spot in those individuals with only unfertilised eggs was consistently pale. Taken together, the increased darkness of the spot with advancing development may imply that it is used to ward off or confuse the males, perhaps by way of mimicking a ‘second’ pair of eyes—protecting both the female and developing young from the potential damages of gonopodial thrust of a mating male. It is well documented that the male poecilids including Gambusia possesses gonopodia with serrated tips, hooks and claws as accessory structures to facilitate coercive copulation [31–33] and cause injuries/bleeding at the female genital pore [34]. Conceivably, the fragile developing embryos are at greater risk of damage should copulation occur during ‘pregnancy’/gravidity. Observation that male Gambusia avoid mating with females closer to parturition [35] also support the notion that the gravid spot serves the purpose of repelling/warding off mating males during the period of gestation -more so closer to parturition, when the developing embryos are most vulnerable.

It is as yet unknown what mechanism/s triggers the progression of melanisation on the egg sac but it is likely linked to physiological responses in sync with elevated steroid hormone levels. For example, several studies have suggested that elevated steroid hormone levels regulate nuptial coloration in fish species such as the medaka (*Oryzias latipes*), guppy (*Poecilia reticulata*) and two-spotted goby (*Gobiusculus flavescenes*) [36–40] as melanophores exhibit higher motility compared to other types of chromatophores when stimulated by nervous or hormone activity i.e. triggering aggregation of melanophores [41]. Nevertheless, further studies are needed to confirm and identify the hormones that might cause the migration, concentration and dispersal of melanophores that regulate the gravid spot colour intensity, during and after gravidity in *G*. *holbrooki* and poecilids in general. Noting that most previous studies [36–40] have focused on pigments located externally on the fish skin, this study should help direct future investigations to internal pigmentation of the embryonic sac.

### Gravid spot can predict developmental progress, clutch size and timing of parturition

As has been demonstrated by this study, the gravid spot in *G*. *holbrooki* can be used to predict the embryonic development, clutch size and timing of parturition in females. Interestingly, the gravid spot appears semi-autonomous in that it does not physically derive its coloration from the developing embryos, but yet displays remarkable association with the progression of development and reproductive output. This established relationship should simplify future studies, both in the field and laboratory particularly in instances where the use of the gravid spot as an external marker could reduce/avoid the need to sacrifice brooding females and facilitate close synchronisation of developmental and reproductive status between individuals as may be critical in most comparative experiments.

Our observation shows that the size and shape of the gravid spot between individuals varies. For example, a smaller gravid female might possess a larger spot compared to a bigger female that may be undergoing recrudescence. This was also quite obvious from observations post-parturition, where the gravid spot decreased in size initially before regaining in size as the female goes through its next reproductive cycle. This is in agreement with observations of Howell, Black [29] in this species where the size of the gravid spot increased with progression of gestation, albeit shrinking and expanding between successive gestation cycles.

As suggested previously [30] the gravid spot is ‘formed’ or more accurately revealed due to the tearing of the peritoneum, its actual shape and size in each individual may be determined by how the peritoneum is ‘ruptured’. Therefore like a fingerprint, the pattern of the gravid spot appears unique to each individual, a feature potentially useful in certain studies for example, in behavioural studies where the identification of individuals without intrusive external marking is important. However, it must be noted that both size and shape of the gravid spot display plasticity associated with swell and shrink of the belly associated with gravidity and recrudescence, respectively.

To our knowledge, this is the first study that quantifies and demonstrates a direct relationship between the intensity and size of the gravid spot, with key developmental and reproductive traits in a livebearing fish. This in part was facilitated by recent advances in digital imaging and analysis [24, 27, 42, 43]. With the development of software such as ‘ImageJ’ [26] and ‘Expertomica Fishgui’ [44], the process of analysing digital images especially in terms of colour intensities has become easier, faster, much more accurate and are also increasingly amenable to automation. In contrast, studies on Gambusia’s anal spot intensity and size [21, 45] that were carried out long ago were done by visual scoring making them prone to subjective error.

The utility of the gravid spot intensity in informing and refining developmental and reproductive studies was immediately apparent from the gestation and parturition experiments conducted as part of this study. Here the selection of the females of comparable reproductive status was facilitated by the intensity of gravid spot. Only females with a gravid spot intensity category of V were chosen and all females parturated within 1–5 days post-transfer to the individual tank providing greater control over design of downstream experiments and their observation in a timely and more organised fashion. In contrast, when chosen randomly we routinely encountered differences in timing of parturition in excess of 10 days between females—delaying timely inferences, prolonged wait between experiments and needless to say extra infrastructure costs including animal rearing and labour associated with the observations. The greater certainty offered by the intensity values in predicting the developmental progress and timing of parturition would assist in improving accuracy in developmental studies. Similarly, improvements in accuracy in pollution exposure studies are also easily conceivable. For example, repeat measure analysis associated with exposure to endocrine disruptors could potentially be quantified more readily, reliably, easily and potentially non-invasively, without the need for sacrificing the animals.

### *G*. *holbrooki* exhibits superfetation during gestation

Scrimshaw [46] reported that almost all poecilid species he examined, including *G*. *holbrooki* exhibit occasional superfetation i.e. the presence of more than one clutch of developing embryos in the same female at the same time, and suggested that it is possibly just a variation within a litter and does not mean that the embryos are from a different litter. It was also suggested that the embryos in the earlier developmental stage are underdeveloped and will be reabsorbed. Several other earlier studies have reported that in the genus Gambusia, superfetation does not commonly occur [46–49]. In the current study, the observation of embryos at more than one development stage together with mature unfertilized eggs in most brooding mothers, suggests that superfetation is a norm and not an exception in this species. Furthermore, in most females the differences between the embryonic stages were quite significant and tended to fall into two distinct stages. For example, in one female, that contained 24 embryos, 19 of them were at stage II while the remaining five were significantly advanced (stage V) and ready for parturition.

Interestingly, observations on the embryonic development in females of wild *G*. *holbrooki* populations in Tasmania [50] did not find any sign of superfetation in this species. Scrimshaw [46] has suggested that superfetation is uncommon in most poecilids but that it can occur under favourable natural environmental conditions or under special laboratory conditions of constant lighting and unlimited food supply. For example, the occurrence of superfetation in least killifish (*Heterandria formosa*) was high during spring and summer but low during autumn and winter while superfetation was high in guppies (*Poecilia reticulata*) exposed to constant artificial light under laboratory conditions. In molly (*Poecilia sphenops*), superfetation is reported to occur only under very favourable environmental conditions and not under captive conditions in the laboratory [46]. On balance, it is likely that this species and perhaps most poecilids adopt a strategy of superfetation to optimise recruitment output when environmental conditions are favourable and default to single clutch gestation, when conditions are less optimal. It is however intriguing as to how they modulate/orchestrate such complex reproductive strategies and respond, for example, to changing environmental climes.

### Gestation is shortened by increased rearing temperature

The current observation shows that temperature has a significant (P<0.05) effect on the gestation period of this species, where a slight increase in temperature (2°C) reduced the gestation period by 26.67%. Conceivably, an increased temperature significantly increased the rate of development resulting in reduced gestation period. The rate of embryonic development in fish in general is known to be significantly influenced by temperature—accelerated with increasing temperature within an optimal range, but retarded as it reaches the upper lethal threshold of any given species (For review see Rombough [51]). The observed disproportional (> twice every 10°C) increase in gestation period at 23°C suggests that the temperature is outside the optimal breeding range for the species and concur with an earlier suggestion that optimum breeding temperature for this species is 25–30°C [50]. The observed average gestation periods (28.9 and 39 days at 25 and 23°C respectively) are comparable to those reported for wild populations (34 days) in Tasmania [50] and concur with observations in the guppy that the gestation is longer (40–60 days) when reared at lower than optimal temperatures (20°C and 23°C) [52].

### *G*. *holbrooki* predominantly parturates in the morning, with babies emerging ‘tail-first’

Diel-timing of parturition in livebearers is known to vary from one species to another. For example, in *G*. *affinis* it is known to occur during early morning [53] while in guppies it is reported to occur in the evening [54]. Our observations show that *G*. *holbrooki* commonly parturates in the morning, very similar to *G*. *affinis*. The diel mechanisms that trigger parturition in each species are somewhat unclear. However, it has been shown that the parturition in *G*. *affinis* can be stimulated by a sudden decrease of water temperature [55], suggesting that dropping water temperature associated with dawn could provide the trigger. Nonetheless, the constant rearing temperature in this study and those of Senior [53], suggests another factor particularly lights might play a greater role in triggering parturition in both *G*. *holbrooki* and *G*. *affinis*.

To our knowledge this is the first study to describe the parturition process in *G*. *holbrooki*. Our observations suggest that ‘tail-first’ birth is the norm in the species, with ‘head-first’ births constituting breech in this species. Similar observations of both head- and tail-first births also occur in the guppy (*Poecilia reticulata*) except that the head-first birth was the most frequent [54]. Earlier studies on *G*. *affinis* have reported that the tail emerged first with no mention of other patterns [6, 54]. The contrasting birthing postures may reflect species-specific reproductive strategies, providing clues for habitat/adaptive diversity and evolutionary relationships among live-bearing fish.

The observation that yolk is completely absorbed at the time of parturition is in agreement with those reported in guppy [56], suggesting that the embryos born with yolk sac still evident are ‘premature’. A similar premature birth condition was observed in guppies and swordtails, where birth of immature embryos and ova alongside live and well-developed fry was attributed to non-functional superfetation [57]. Thus, superfetation may in part explain the occurrence of premature birth in a few *G*. *holbrooki* females observed in this study. However, this is unlikely as in at least three females the entire clutch of fry was born prematurely and survived except for a few that were born with burst yolk sac. Reports in mollies and guppies have attributed handling stress as the main reasons for premature births [58, 59]. It is more likely, the process of transfer of brood fish from stock to breeding tanks may have caused stress in the three pregnant females causing them to give birth prematurely.

## Conclusions

This study demonstrates that the gravid spot can be used to predict the embryonic development, timing of parturition and clutch size of *G*. *holbrooki* facilitating better design and observation of downstream experiments. Predictably, the gestation period in this species is significantly influenced by temperature and parturition takes place mainly in the morning. The study also shows that the parturition in *G*. *holbrooki* occurs predominantly ‘tail first’ with few exceptions of ‘head-first’, twin and premature births. Future investigations on basic reproductive biology, including mechanisms of gravid spot melanisation, superfetation, sperm activation and triggers of parturition in this species as well as their utilisation in applied pollution research and management of pest populations will be greatly facilitated by the methods, observations and relationships established in this study.

## Supporting Information

S1 Table Summary of multiple regression analysis. (DOCX)

S1 Video Parturition postures of *G*. *holbrooki*. (ZIP)
