# Supplementary material for: Gravid Spot Predicts Developmental Progress and Reproductive Output in a Livebearing Fish, Gambusia holbrooki
Source: PLoS One. 2016 Jan 25;11(1):e0147711. doi: 10.1371/journal.pone.0147711 (PMC4725957; doi:10.1371/journal.pone.0147711)
Supplement: S1 Table — (DOCX) [file pone.0147711.s001.docx]

**S1 Table. Summary of multiple regression analysis**

| Variable | B | SE_B_ | β |
| --- | --- | --- | --- |
| Intercept | 1.835 | 0.704 |  |
| Intensity | -0.85 | 0.317 | -0.365* |
| Fish Length | 0.196 | 0.084 | 0.295* |
| Spot Size | 3.549 | 3.130 | 0.155* |

Note: *p<0.05, B= unstandardized regression coefficient; SE_B_= Standard error of the coefficient; β= standardized coefficient
